# Effect of progesterone infused controlled internal drug releasing (CIDR) device and timing of gonadotropin stimulation using P.G. 600 on reproductive success in ewes bred out of season

**DOI:** 10.1093/tas/txad081

**Published:** 2023-07-19

**Authors:** Reid R Redden, Tammi L Neville, Danielle N Black, Mellissa R Crosswhite, Crosswhite Carl R Dahlen

**Affiliations:** Center for Nutrition and Pregnancy and Department of Animal Sciences, North Dakota State University, Fargo, USA; Texas A&M AgriLife Research & Extension Center and Department of Animal Science, Texas A&M University, San Angelo, TX, USA; Center for Nutrition and Pregnancy and Department of Animal Sciences, North Dakota State University, Fargo, USA; United States Department of Agriculture—NIFA, Kansas City, MO, USA; Center for Nutrition and Pregnancy and Department of Animal Sciences, North Dakota State University, Fargo, USA; Center for Nutrition and Pregnancy and Department of Animal Sciences, North Dakota State University, Fargo, USA; Department of Animal and Food Sciences, Oklahoma State University, Stillwater, OK, USA; Center for Nutrition and Pregnancy and Department of Animal Sciences, North Dakota State University, Fargo, USA

**Keywords:** gonadotropins, out of season breeding, progesterone, sheep, synchronization

## Abstract

Objectives of this study were to determine effects of exogenous progesterone (via controlled internal drug releasing devices; CIDR) in combination with exogenous gonadotropins (PMSG/hCG) use either at CIDR removal or 1 d before CIDR removal to induce estrus and cyclicity and subsequently enhance the proportion of ewes lambing, lambing rate, prolificacy, and days to lambing in ewes bred out of season. Multiparous ewes (*n* = 414) were randomly assigned to one of four treatments: untreated (**U**, *n* = 122), 7 d CIDR (**C**, *n* = 97), 7 d CIDR plus P.G. 600 (240 IU pregnant mare serum gonadotropin [PMSG] and 120 IU human chorionic gonadotropin [hCG]) at CIDR removal (**CPG0**, *n* = 97), and 7 d CIDR plus P.G. 600 (240 IU PMSG and 120 IU hCG) 1 d before CIDR removal (**CPG-1**, *n* = 98). Rams (*n* = 15) were joined with ewes immediately after CIDR removal and remained with ewes for a 21 d breeding period. Lambing data were summarized for the first 10 days of the lambing season and overall. Categorical data were analyzed using the GLIMMIX procedure of SAS whereas non-categorical data were analyzed using the mixed procedure. Proportion of ewes lambing in the first 10 d was greater (*P* < 0.05) for CPG0 and CPG-1 ewes compared with C ewes, which was greater (*P* < 0.0001) compared with U ewes. Overall proportion of ewes lambing was greater (*P* ≤ 0.0001) in all treatments utilizing CIDR compared with U ewes, but no differences (*P* ≥ 0.84) due to P.G. 600 were detected compared with C. Lambing rate in the first 10 d was greater (*P* < 0.05) for CPG-1 than C, with CPGO being intermediate, and all CIDR-treated ewes being greater than U (*P* < 0.0001). Overall lambing rate increased (*P* ≤ 0.0001) in all treatments utilizing CIDR compared with U ewes, but no differences (*P* ≥ 0.76) due to P.G. 600 were detected compared with C. Prolificacy was similar among all treatments both for the first 10 d of the lambing season (*P* = 0.86) and overall (*P* = 0.80). Day of lambing in the lambing season was reduced (*P* ≤ 0.03) for CPG0 and CPG-1 compared with CIDR-treated ewes, which was reduced (*P* < 0.0001) compared with U ewes (days 10.6, 9.0, 13.4, and 24.4 ± 0.9 for CPG0, CPG-1, CON, and U, respectively). Though timing of P.G. 600 did not influence results, the combination of CIDR and P.G. 600 enhanced the proportion of lambs born earlier in the lambing season, and incorporating a CIDR with or without P.G. 600 enhanced the overall proportion of ewes lambing and lambing rate in out-of-season breeding scenarios.

**Implications**

The combinations of CIDRs and PG600 used in this experiment successfully induced estrus and cyclicity in a portion of ewes in the out of season breeding system evaluated. Days to lambing were decreased by 13 d with the use of CIDR, and further decreased by 3.5 d with the use of P.G. 600. In the first 10 d of the lambing season, lambing rate was increased over untreated ewes by 40% with the use of CIDR, which was further increased by another 12% to 17 % with the use of P.G. 600. Our observations of similar reproductive outcomes between ewes treated with P.G. 600 at the time of CIDR removal and 1 d prior indicate that producers can reduce labor required for handling ewes during synchronization by administering P.G. 600 concurrently with CIDR removal. Therefore, the combination of CIDR and P.G. 600 (3 mL, containing 240 IU PMSG and 120 IU hCG) may be beneficial in increasing the number of ewes lambing and increasing the number of lambs born in the first 10 d of lambing season in natural service breeding systems with no pre-breeding male exposure, resulting in increased overall productivity in an out of season breeding scenario.

## Introduction

The seasonal breeding pattern of sheep presents challenges for producers wishing to target lambing dates outside of those that align with natural seasonality patterns. The transition out of the anestrous period involves progesterone priming of the brain as a prerequisite for estrous cycles of normal length (Rosa and Bryant, 2003). Exogenous progesterone administered in the form of controlled internal drug releasing (CIDR) devices has been shown to induce ewe estrus during the anestrous period (Knights et al., 2001; Habeeb and Kutzler, 2021; Hameed et al., 2021). In 2009, CIDR sheep inserts were approved for use to induce estrus in seasonally anestrous ewes by the United States Food and Drug Administration, and researchers have identified that net profit was maximized when incorporating a CIDR for a 6 d synchronization period (vs. 3, 9, and 12 d) during the breeding season (Swelum et al., 2018). However, overall fertility of ewes treated with CIDRs during the anestrous period has been less than fertility of ewes during the normal breeding season (Crosby et al., 1991; Habeeb and Kutzler, 2021). As a result, cost-effectiveness of this tool has been less than ideal for commercial sheep producers.

Exogenous gonadotropins are commonly administered at CIDR removal to induce ewe estrus during the anestrous season. A commonly used product, P.G. 600, is a mixture of equine chorionic gonadotropin (also called pregnant mare serum gonadotropin (PMSG), with FSH- and LH-like activity) and human chorionic gonadotropin (LH-like activity) and is approved in the United States for inducing estrus in swine (Britt et al., 1989). Limited data are available on the efficacy of P.G. 600 with regard to timing of use (Safranski et al., 1992; Jabbar et al., 1994; Windorski et al., 2008) and the effects of P.G. 600 around the time of short-term CIDR removal on reproductive efficiency in out of season breeding scenarios. Jackson et al. (2014) demonstrated fewer days to estrus using 5-d CIDR and 5-d CIDR plus GnRH (0.025 mg gonadorelin hydrochloride) at CIDR removal compared with untreated control ewes during the normal breeding season. Data from D’Souza et al. (2014) showed P.G. 600 (3 mL, containing 240 IU PMSG and 120 IU hCG) administered at CIDR removal (at day 5) did not improve fertility compared to CIDR alone; but, when P.G. 600 was administered 1 d prior to CIDR removal (at 5 d) both pregnancy and prolificacy rate were improved compared with CIDR alone in anestrous ewes. However, data comparing the effects of a CIDR with P.G. 600 administered at the time of CIDR removal or 1 d before removal within the same experiment are lacking.

Therefore, the objectives of this study were to evaluate the effects of using exogenous progesterone (in the form of a CIDR) alone for 7 d, or in combination with exogenous gonadotropins (PMSG and hCG in the form of P.G. 600) 1 d before or at the time of CIDR removal, on reproductive performance of ewes bred out of season. Our hypothesis was that including a CIDR to induce cyclicity in ewes bred out of season by natural service rams would increase the proportion of ewes lambing and the number of lambs born per ewe compared and decrease average day of birth in the lambing season compared with untreated ewes and that inclusion of P.G. 600 1 d before or at the time of CIDR removal would further enhance these metrics.

## Material and Methods

Protocols described herein were approved by the North Dakota State University Institutional Animal Care and Use Committee (Protocol A14065).

This study was conducted on a commercial sheep operation in central North Dakota (Approximate latitude: N 46° 52ʹ 30.7683″). Multiparous western white-faced crossbred ewes (mainly crosses of Rambouillet, Targhee, Columbia, and/or Merino; *n* = 414) were all managed similarly for the duration of the research project. During the breeding period, ewes were grazing native prairie grass as a single group.

**Figure 1.**
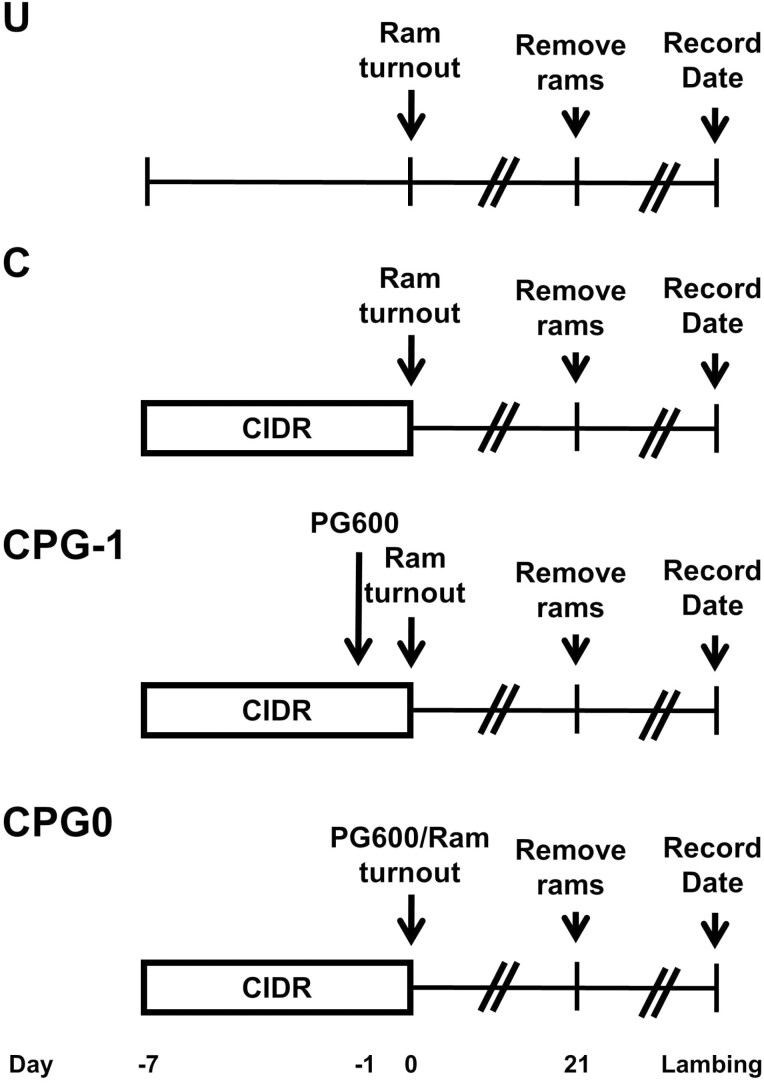
Schematic of treatments applied to crossbred multiparous ewes to synchronize out of season breeding. U—untreated control; C—received CIDR for 7 d; CPG0—received a CIDR for 7 d plus 3.0 mL P.G. 600 (240 IU PMSG and 120 IU hCG) at removal; CPG-1- received CIDR for 7 d plus 3.0 mL P.G. 600 (240 IU PMSG and 120 IU hCG) 1 d prior to CIDR removal.

In late July, all ewes received a project ID ear tag in each ear and were assigned a body condition score (2.64 ± 0.018 on a scale of 1 to 5; McHugh et al., 2018) by a trained technician. Ewes were randomly assigned to one of four treatments (Figure 1): 1) untreated (**U**, *n* = 122); 2) 7 d CIDR insert (EAZI-BREED CIDR Sheep Insert, 0.3 g P4, Zoetis Animal Health, Florham Park, NJ; **C**; *n* = 97); 3) 7 d CIDR plus P.G. 600 (3 mL, containing 240 IU PMSG and 120 IU hCG); Merck Animal Health, Intervet Inc. Madison, NJ at removal (**CPG0**, *n* = 97); or 4) 7 d CIDR plus P.G. 600 (3 mL, containing 240 IU PMSG and 120 IU hCG) 1 d before CIDR removal (**CPG-1**, *n* = 98). The dose of P.G. 600 used in the current study was in the range of optimal dosage for anestrous ewes (between 160 and 280 IU PMSG and 80 and 140 IU hCG, respectively; Cross et al., 2019). Ewes had no exposure to rams before the breeding season to control any confounding influence of the male effect. Rams (*n* = 15) were joined with ewes immediately after CIDR removal and remained with ewes for a 21 d breeding period.

Ewes were monitored for lambing and the date of birth and number of lambs were recorded for each ewe. Resultant data served as proxy measures for hastening cyclicity in out of season breeding and subsequent calculations included: 1) Ewes lambing: number of ewes lambing/ewes treated; 2) Lambing rate: number of lambs/ewes treated; 3) Prolificacy: number of lambs/ewes lambing.

Data for proportion of ewes lambing, lambing rate, and prolificacy were further classified to represent ewes that lambed in the first 10 d of the lambing season (i.e., the synchronized breeding period) and all ewes that lambed (i.e., overall). Data for date of birth were evaluated considering day 1 as the day the first live lamb was born and each consecutive date being a consecutive day in the lambing season. Data for date of birth in the lambing season could also be interpreted as the day during the breeding season which the ewe theoretically conceived, with smaller numbers indicating that the ewes conceived earlier in the breeding season.

The mixed procedure of SAS (SAS Inst. Inc., Cary, NC) was used to analyze non-categorical data (lambing rate, prolificacy, day of birth in the lambing season), and procedure GLIMMIX was used to analyze categorical data (proportion of ewes lambing). Treatment was included as fixed effects in the model and body condition score was included as a covariate. Means were separated by using the PDIFF function of SAS and differences were determined at *P* ≤ 0.05.

The LIFETEST procedure of SAS was used to calculate Kaplan–Meier survival estimates for lambing distribution. Data are presented as an accumulated proportion of lambs born over time (i.e., charted as failures rather than survival). A Bonferroni-corrected log-rank test was used to separate survival means for pair-wise comparisons and differences were considered significant at *P* ≤ 0.05. Lambing distribution data were also analyzed using PROC PHREG to evaluate the Cox proportion hazard. Effects are expressed as relative hazard ratios using the CPG0 treatment as the reference value, so values greater than 1 indicate greater risk of lambing earlier in the lambing season whereas values below 1 indicate greater risk of lambing later in the lambing season.

## Results and Discussion

The exogenous progesterone and gonadotropins used in this experiment successfully initiated estrus and cyclicity in a portion of ewes as evidenced by the proportion of ewes lambing and the patterns of lambing data observed. Treatment with CIDR alone or in combination with P.G. 600 resulted in a greater proportion (*P* < 0.001) of ewes lambing compared with untreated ewes (Table 1). Similarly, Safranski et al. (1992) reported an increase in the proportion of ewes lambing with the use of MGA (similar mode of action to CIDR) alone or in conjunction with P.G. 600, and pregnancy rates to both a synchronized estrus and the subsequent return breeding were greater in ewes treated with either a CIDR or a progestagen-impregnated sponge compared with untreated ewes (Martinez-Roz et al., 2018). In addition, Knights et al. (2015) observed improvements in pregnancy rate and proportion of ewes lambing when fall born ewes lambs received P.G. 600 at CIDR removal in a 5-d program, but saw no effect in yearling nulliparous ewes. Though D’Souza et al. (2014) observed increased proportions of ewes lambing in an experiment when P.G. 600 was administered 1 d before CIDR removal and no effect in a different experiment when P.G. 600 was administered at the time of CIDR removal, our model included both treatments (P.G. 600 at the time of, and 1 d before CIDR removal) within the same experiment and revealed no differences in overall proportion of ewes lambing between ewes receiving P.G. 600 at CIDR removal or 1 d before (*P* ≥ 0.84). Our results indicate that producers can use CIDR inserts in 7 d protocol in multiparous ewes with or without P.G. 600 at a 3 mL dose (240 IU PMSG and 120 IU hCG) to enhance the proportion of ewes that lamb in an out-of-season natural mating scenario. In addition, producers can the reduce labor associated with sheep gathering and handling by administering P.G. 600 concurrently with CIDR removal rather than adding an additional handling event solely to administer P.G. 600.

**Table 1.** Effect of progesterone infused controlled internal drug releasing device (CIDR) and timing of gonadotropin stimulation using P.G. 600 on lambing rate and prolificacy in ewes bred out of season

	Treatments^1^					
Item	U	C	CPG-1	CPG0	SEM	*P*-Value
Ewes lambing, n (%)^2^						
First 10 d	1/122 (0.8)^a^	41/97 (42.3)^b^	58/98 (59.2)^c^	53/98 (54.1)^c^	4.25	<0.0001
Overall	60/122 (49.2)^a^	71/97 (73.2)^b^	73/98 (74.5)^b^	71/98 (72.5)^b^	4.67	<0.0001
Lambing rate, no.^3^						
First 10 d	0.01^a^	0.73^b^	1.02^c^	0.91^bc^	0.08	<0.0001
Overall	0.85^a^	1.25^b^	1.23^b^	1.19^b^	0.09	<0.0001
Prolificacy, no.^4^						
First 10 d	2.00	1.78	1.74	1.68	0.63	0.86
Overall	1.73	1.70	1.67	1.63	0.08	0.80

^1^Treatments: untreated (U), 7 d CIDR (C), 7 d CIDR plus P.G. 600 (240 IU PMSG and 120 IU hCG) at removal (CPG0), and 7 d CIDR plus P.G. 600 (240 IU PMSG and 120 IU hCG) 1 d prior to CIDR removal (CPG-1).

^2^Ewes lambing = proportion of ewes lambing per ewe treated.

^3^Lambing rate = number of lambs born per ewe treated.

^4^Prolificacy = number of lambs born per ewe lambing.

^a,^ ^b,^ ^c^Means within row lacking common superscript differ (*P* < 0.05).

Using P.G. 600 in combination with a CIDR (CPG0 and CPG-1 treatments) resulted in a greater proportion (*P* < 0.05) of ewes lambing in the first 10 d of the lambing season compared with CIDR alone, which had more (*P* < 0.001) ewes lambing in the first 10 d of the lambing season compared with untreated ewes in a natural mating scenario. The effects of using a CIDR to enhance the proportion of ewes bred early in the breeding season compared with untreated ewes have been shown by numerous researchers (Rosasco et al., 2019; Habeeb and Kutzler, 2021; Hameed et al., 2021). Results of efforts evaluating the impacts of PG 600 on the proportion of ewes lambing in the first 10 d of the lambing season (i.e., reflective of becoming pregnant to the synchronized breeding) are inconsistent. For example, though enhanced pregnancy rate and proportion of ewes lambing to the synchronized breeding were observed in fall born ewe lambs after addition of P.G. 600 to a CIDR protocol, a group of yearling ewes actually had a decreased proportion of ewes lambing to 1st service breeding compared with using a CIDR alone (Knights et al., 2015). Some reductions in reproductive performance observed in other experiments are likely due to dose of P.G. 600 administered. An effort to characterize the impact of a common dose of P.G. 600 (5 mL, also the labeled dose for gilts and sows) revealed a decrease in pregnancy rates in ewes receiving 5 mL (400 IU PMSG and 200 IU hCG) compared with those receiving 1.5 mL (120 IU PMSG and 60 IU hCG; Habeeb et al., 2019). The dose of P.G. 600 used in the current experiment (3 mL, 240 IU PMSG, and 120 IU hCG) falls within the range of the optimal dose identified for anestrous ewes (2.0 to 3.5 mL or between 160 and 280 IU PMSG and 80 and 140 IU hCG, respectively; Cross et al., 2019), and using greater doses within the 7 d CIDR protocol tested in the current experiment could compromise pregnancy rates.

Prolificacy (i.e., number of lambs born per lambing) was similar between all treatments both for the first 10 d of the lambing season (*P* = 0.86) and overall (*P* = 0.80). Other researchers (Safranski et al., 1992; Jabbar et al., 1994; Windorski et al., 2008; Jackson et al., 2014; Knights et al., 2015) also noted no differences in prolificacy after treatment with progesterone or progesterone-like products, or with the addition of P.G. 600 to progestin-based protocols. However, D’Souza et al. (2014) did observe a tendency for increased prolificacy when P.G. 600 (240 IU PMSG and 120 IU hCG) was administered at the time of CIDR removal, but not when administered 1 d before removal. As the dose of P.G. 600 increases, so too does the incidence of multiple ovulations, which would drive increased prolificacy (Cross et al., 2019; Habeeb et al., 2019), but this enhanced prolificacy comes at the expense of reduced pregnancy rates. Based on our results we would not anticipate an improvement in out-of-season breeding prolificacy after CIDR administration, with or without administrations of 3 mL P.G. 600 (240 IU PMSG and 120 IU hCG).

**Figure 2.**
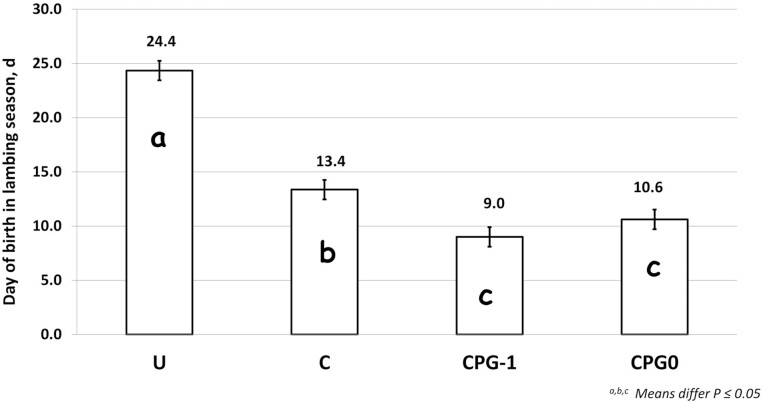
Effect of progesterone infused controlled internal drug releasing device (CIDR) and timing of gonadotropin stimulation using P.G. 600 on day of birth in the lambing season in ewes bred out of season. Treatments: untreated (**U**, *n* = 122), 7 d CIDR (**C**, *n* = 97), 7 d CIDR plus 3.0 mL P.G. 600 (240 IU PMSG and 120 IU hCG) at removal (**CPG0**, *n* = 97), and 7 d CIDR plus 3.0 mL P.G. 600 (240 IU PMSG and 120 IU hCG) 1 d prior to CIDR removal (**CPG-1**, *n* = 98). ^a, b, c^ means differ *P* ≤ 0.03.

**Figure 3.**
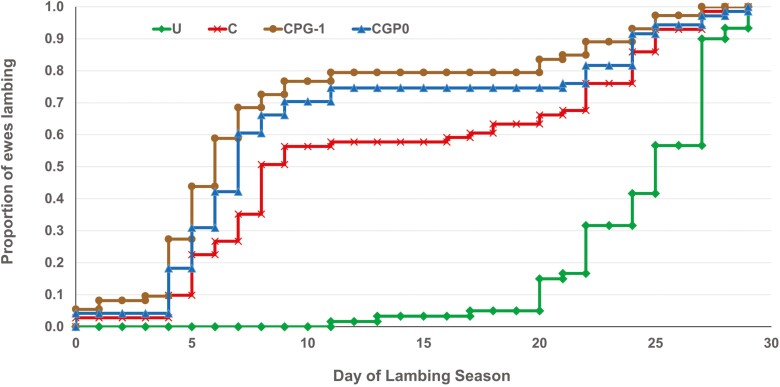
Survival curves representing the day of the lambing season for ewes exposed to progesterone-infused controlled internal drug releasing device (CIDR) with and without P.G. 600. Treatments: untreated (**U**, *n* = 122), 7 d CIDR (**C**, *n* = 97), 7 d CIDR plus 3.0 mL P.G. 600 (240 IU PMSG and 120 IU hCG) at removal (**CPG0**, *n* = 97), and 7 d CIDR plus 3.0 mL P.G. 600 (240 IU PMSG and 120 IU hCG) 1 d prior to CIDR removal (**CPG-1**, *n* = 98).

Calculation of data of birth in the lambing season revealed that lambs from ewes in the CIDR treatment were born 11 d earlier (P< 0.001) compared with untreated ewes (Figure 2) and that adding P.G. 600 to the CIDR treatment (CPG0 and CPG-1 treatments) resulted in lambs being born an additional 3 to 4 d earlier (*P* ≤ 0.03) in the lambing season compared with CIDR treatment alone. In addition, pairwise comparisons of survival curve data (Figure 3) corroborated the calculated values for lambing distribution, showing that lambs born from untreated ewes (24 ± 0.48 d) had greater (*P* < 0.001) time to lambing that all ewes receiving a CIDR, and that ewes from the C treatment (13.4 ± 1.04 d) had greater (*P* = 0.05) time to lambing than CPG-1 ewes (8.97 ± 0.90 d), with CPG0 ewes being intermediate (10.62 ± 1.04 d; *P* ≥ 0.53). Furthermore, the estimated hazard ratios for the day of birth in the lambing season with 95% confidence intervals (*P* < 0.001) were 0.34 (0.24 to 0.48) for untreated ewes, 0.83 (0.59 to 1.15) for C ewes, and 1.34 (0.99 to 1.19) for CPG-1 ewes, with CGP0 ewes serving as the reference comparison. Similarly, our lab previously observed that ewes in the transition period (August through October, after the summer solstice) treated with 5 d CIDR lambed 9 d earlier in the lambing season compared to untreated ewes (Jackson et al., 2014), but P.G. 600 administration was not evaluated. Safranski et al. (1992) and Windorski (2008) noted decreased days to lambing with the use of Melengestrol Acetate (**MGA**) either in conjunction with P.G. 600 or alone. However, when D’Souza et al. (2014) evaluated the addition of P.G. 600 at the time of, or 1 d before CIDR removal in a 5 d protocol they did not observe an overall advantage in lamb age compared with CIDR treatment alone. Our results indicate a clear advantage in lamb age and the proportion of lambs born early in the lambing season after CIDR administration to multiparous ewes and an additional advantage when 3 mL P.G. 600 (240 IU PMSG and 120 IU hCG) is incorporated with a 7-d CIDR protocol in an out-of-season breeding scenario.

An additional observation from our survival analysis dataset is a clear demonstration of the ram effect (Fabre-Nys et al., 2015). Treatments with ewes receiving a CIDR had a first bout of lambing, likely as a result of a synchronized estrus, which started in earnest 4 d after the first lambs were born. This initial bout of lambing in ewes receiving CIDRs lasted for 7 d, and no untreated ewes were lambed during this period. Thereafter, was a period of 9 d during which very little lambing occurred for ewes in any of the respective treatments. Forthwith, a large proportion of untreated ewes began lambing, as well as additional ewes exposed to treatments including a CIDR. The ram effect elicits a pulse of LH and increases pulse frequency to culminate in an LH surge within the following 2 to 3 d after ram introduction (Fabre-Nys et al., 2015). As a period of elevated progesterone is often required for females to demonstrate true estrus, the first postpartum ovulation often occurs without estrus (so-called silent heat), and the subsequently formed CL may be either of normal functionality, or short-lived (Rosa and Bryant, 2003). The resultant pattern of estrus response is normal estrus behavior occurring in the interval of 17 to 25 d after ram introduction (Ungerfeld et al., 2003) . The progesterone present in the CIDR in the current experiment likely mimicked the increase in progesterone requisite for a cycle of normal length and allowed subsequent estrus, receptivity to mating, and ovulation to occur in the days immediately following CIDR removal. In addition, perhaps by using a 21 d breeding season in the current experiment, an opportunity for increased numbers of lambs born was missed, as a portion of ewes that experienced a silent estrus and sub-functional luteal status would likely be returning in estrus only 24 d after ram exposure (Ungerfeld et al., 2003).
